# Evaluation of Retinal and Posterior Segment Vascular Changes Due to Systemic Hypoxia Using Optical Coherence Tomography Angiography

**DOI:** 10.3390/jcm13226680

**Published:** 2024-11-07

**Authors:** Nadav Levinger, Nir Erdinest, Ayman Abu Rmeileh, Eisa Mouallem, Shadi Zahran, Sheer Shabat, Yotam Kolben, Talmon Aviv, Rottem Kuint, Liran Tiosano, Samer Khateb

**Affiliations:** 1 Department of Ophthalmology, Hadassah University Medical Center, Faculty of Medicine, The Hebrew University of Jerusalem, Jerusalem 91120, Israel; 2 Institute of Pulmonary Medicine, Hadassah University Medical Center, Faculty of Medicine, The Hebrew University of Jerusalem, Jerusalem 91120, Israel; 3 Internal Medical Daycare, Hadassah University Medical Center, Faculty of Medicine, The Hebrew University of Jerusalem, Jerusalem 91120, Israel; 4 Department of Medicine B, Hadassah University Medical Center, Faculty of Medicine, The Hebrew University of Jerusalem, Jerusalem 91120, Israel; 5 Department of Physical & Medical Rehabilitation, Hadassah University Medical Center, Faculty of Medicine, The Hebrew University of Jerusalem, Jerusalem 91120, Israel; 6 Department of Medicine A, Hadassah University Medical Center, Faculty of Medicine, The Hebrew University of Jerusalem, Jerusalem 91120, Israel; 7 Department of Medicine C, Hadassah University Medical Center, Faculty of Medicine, The Hebrew University of Jerusalem, Jerusalem 91120, Israel

**Keywords:** hypoxic lung disease, retinal capillary density, optic nerve head, choriocapillaries, chronic and acute-on-chronic hypoxia, optical coherence tomography angiography, choroidal capillaries

## Abstract

**Background/Objectives:** Retinal vascular occlusions are a significant cause of visual impairment in older adults, resulting in ischemic retinal damage and sudden vision loss. This study evaluates the retinal, optic nerve head (ONH), and choroidal capillary networks in chronic and acute-on-chronic hypoxia compared to normal controls using optical coherence tomography angiography (OCT-A). **Methods:** We evaluated a prospective study including twenty patients in the hypoxic group (mean age 61.2 ± 10.2) in two phases, chronic hypoxia and acute-on-chronic hypoxia, and 21 control subjects (mean age 59 ± 9.4 years). All patients underwent a comprehensive eye examination, OCT, and OCT-A imaging. The data were analyzed using OCT-A analysis software (Zeiss OCT-A software 2.1.0.55513) and Fiji software (1.51a). Vascular density of the retina and ONH, choriocapillaries, and foveal avascular zone (FAZ) size were measured. **Results:** The superficial peripapillary vascular density was higher for the control group (0.387 ± 0.03) compared to the hypoxic patients with (0.383 ± 0.03) and without O2 supplementation (0.383 ± 0.03; *p* = 0.018). No retinal angiographic differences were identified between the two study groups. The ganglion cell layer (GCL) was thinner in the hypoxic group. Both hypoxic subgroups demonstrated denser choriocapillaries (mean 13,073 ± 1812 and 12,689 ± 1815, with and without O2 supplementation, respectively) compared to the control group (mean 9749 ± 2881, *p* < 0.001 for both groups). Hypoxic patients demonstrated increased area size of choriocapillaries (+O2 supplementation—mean 44,347 ± 10,563; −O2 supplementation—mean 46,984 ± 12,822) compared to the control group (mean 30,979 ± 9635; *p* < 0.01 and *p* < 0.001, respectively). **Conclusions:** Chronic and acute-on-chronic hypoxia did not affect the retinal vascular network, most probably due to the strong autoregulation of vascular function of the retina. However, compared to the control group, GCL, ONH vasculature density, and most choriocapillaries indices were significantly altered among hypoxic patients.

## 1. Introduction

The advent of optical coherence tomography has profoundly transformed the practice of ophthalmology [1]. In recent years, OCT angiography (OCT-A) has emerged as a powerful non-invasive imaging tool to evaluate the microvasculature of the retina and choroid [2]. Imaging retinal capillaries is unique to the human body, enabling systemic disease monitoring.

The vascular network depicted in OCT-A reflects blood motion within the retinal vessels, utilizing intrinsic signals that may be altered by insufficient blood supply [2]. Potential causes include various systemic diseases such as diabetic retinopathy [3], central and branch retinal vein occlusions [4], congestive heart failure [5], atherosclerosis, and many other retinal vascular diseases [2,6]. Previous structural OCT studies have shown thinning of the nasal retinal nerve fiber layer (RNFL), global ganglion cell layer (GCL) loss [7], and a thinned choroid layer in patients with chronic obstructive pulmonary disease (COPD), as low hypoxic levels may damage the choroidal structure [8]. Recently, OCT-A imagery detected differences in the vascular capillary network in children with obstructive sleep apnea (OSA) compared to healthy subjects [9]. The indices of the parafoveal vessels, including vascular diameter, vascular area density, vascular skeleton density, the vessel perimeter index, and the foveal avascular zone, were significantly lower in the OSA group compared to the control group [9].

Chronic hypoxia systematically affects the vasculature of various organs [10,11]. Recently, Songur et al. showed an enlargement of the FAZ and a decrease in the capillary density of the superficial (SCP) and deep capillary plexuses (DCPs) [12]. Alterations in retinal veins were reported but not in arteriolar networks, which may be due to the augmented autoregulation of the arteries compared to the increased resistance of the pulmonary circulation system [12,13]. It is unclear whether COPD and chronic hypoxia affect the choroid’s thickness and capillary density in the peripapillary and macular areas, as studies have presented variable and inconsistent findings [7,13,14].

The current prospective study investigates the impact of chronic and acute-on-chronic hypoxia, resulting from various hypoxic conditions, on the structural and vascular properties of the retina, choroid, and optic nerve head, as assessed by OCT-A, in comparison to an age-matched control group.

## 2. Materials and Methods

### 2.1. Study Design and Patient Cohort

A prospective, single-center study was conducted per the tenets of the Declaration of Helsinki and was approved by the institutional ethics committee of the Hadassah-Hebrew University Medical Center (0824-20-HMO). All subjects signed an informed consent form prior to their participation. Patients aged 40 to 80 years old were recruited from the internal medicine daycare unit and pulmonology clinic; patients diagnosed with COPD or other hypoxic lung diseases at the Hadassah-Hebrew University Medical Center, Jerusalem, Israel, were admitted. All patients were considered stable without acute respiratory exacerbation at the time of recruitment. However, following the study protocol, all patients experienced acute-on-chronic hypoxia as they were examined without supplemental O2, leading to reduced O2 saturation. Patients were divided into two groups: 34 patients were included in the study group with chronic hypoxia due to different diseases, including COPD, idiopathic pulmonary fibrosis, pulmonary hypertension, post-COVID-19 lung disease, and restrictive lung disease (Supplementary Table S1). The second group included 23 age-matched control subjects without lung disease. Patients who did not meet the inclusion criteria were excluded from both groups (Supplementary Figure S1), as 20 patients were included in the hypoxic group and 21 healthy subjects in the control group in the final analysis (Supplementary Table S1 and Supplementary Figure S1). Chronic hypoxia was determined as peripheral blood O2 saturation lower than 92% in room air measured by pulse oximeter. Systemic inclusion criteria other than hypoxia for the study group were the chronic use of supplemental O2 for more than six months before recruitment and stable disease at the time of examination. Patients with ocular diseases, including glaucoma, retinal detachment, diabetic retinopathy, age-related macular degeneration, previous retinal surgeries, or any other ocular conditions suspected to affect the study results per the researchers’ evaluation, were excluded (see Supplementary Data).

All patients underwent a comprehensive ocular exam, including best-corrected visual acuity (BCVA) in decimal units, axial length measurement using IOLMaster^®^ 700 (Carl Zeiss Meditec, Jena, Germany), biomicroscopy, tonometry using iCare (iCare IC200, Helsinki, Finland), and a dilated fundoscopy. Data collection classifications included demographics, comorbidities, ocular history, and COPD Global Initiative for Chronic Obstructive Lung Disease (GOLD). In addition, previous treatment for COPD medications was collected, and the patients filled out the COPD Assessment Test (CAT) questionnaire.

The study utilized OCT angiography imaging to investigate ocular parameters across three distinct regions. Specifically, the optic nerve head was assessed for capillary density, perfusion, and retinal nerve fiber layer thickness. Additionally, vascular parameters of the retina were analyzed, including capillary density, perfusion, and retinal thickness at multiple levels of the retinal layers. Furthermore, the choriocapillaries were examined, with the analysis encompassing choroid count, total area, average size, and area percentage. These comprehensive OCT-A assessments provided valuable insights into the structural and vascular characteristics of the retina, optic nerve head, and choroid, which may be altered by systemic hypoxia. The data were examined using OCT-A analysis software Zeiss OCT-A software 2.1.0.55513) and Fiji image processing software (1.51a).

### 2.2. Image Acquisition

All patients underwent SS-OCTA imaging using the PLEX Elite 9000 device (Carl Zeiss Meditec Inc., Dublin, CA, USA). Macular images of both eyes were obtained using OCT to exclude eyes with additional retinal pathologies. OCT-A imaging of the macula was obtained after dilation with Tropicamide 0.5% drops using a 6 × 6 mm area centered on the fovea and ONH (500 A-scans × 500 B-scans). Imaging was repeated until two OCT-A volume scans were obtained with sufficient image quality (signal strength index > 7) that fulfilled the inclusion criteria (e.g., absence of motion artifact or shadows) as reported previously [15]. A second set of OCT-A images was obtained for the hypoxic group without supplemental O2, performed when O2 saturation was ≤80% or five minutes following disconnecting them from the supplemental O2. Retinal and ONH OCT-A images were analyzed using algorithms in artificial retinal imaging (ARI network; see Supplementary Data). All images were captured during the clinic’s morning hours to avoid diurnal variation.

The manufacturer’s complex optical microangiopathy algorithm was utilized to generate the motion signal by analyzing the variation in intensity and phase between successive B-scans at the same location. The manufacturer’s fully automated retinal layer segmentation algorithm was applied to the three-dimensional structural OCT data segment of the continuous casting (CC) slab as defined previously (10 μm thick starting 31 μm posterior to the RPE-band center line) [16]. The RPE band reference was manually adjusted in all B-scans, in which the fully automated algorithm failed to select the correct segmentation and applied to OCT-A flow intensity and structural data to obtain vascular and structural images of the choriocapillaries, respectively. Of note, the anatomic position of the slab is presumed to be below the anatomic location of the CC and to capture the projection artifact of the CC similarly [15,16]. En-face slabs of the CC were generated from both the flow and structural data in the OCT-A images to allow subsequent signal loss compensation using FIJI software (an expanded version of ImageJ software 1.51a, available at Fiji.sc, National Institutes of Health, Bethesda, MD, USA).

### 2.3. Signal Compensation on OCT-A En-Face CC Images

The inverse of the en-face structural OCT image derived from the slab at the same level as the CC en-face flow image was multiplied with the CC en-face flow image using the previously described method to compensate for potential signal loss below structurally shadowing locations [17].

### 2.4. Computation of CC Flow Deficit Percentage

The resultant en-face compensated CC image (1024 × 1024 pixels) was binarized for quantitative analysis of CC flow deficit % using the Phansalkar method (90.82 µm radius) [18,19]. This radius was chosen to maintain consistency with prior reports [20,21,22]. The image was processed with the “Analyze Particle” command to calculate the signal deficit as a percentage of the 6 × 6 mm macular area. As previously described, the CC directly beneath major superficial retinal vessels was excluded from the analysis to eliminate potentially confounding shadow or projection artifacts. We designed the plugin to measure the above.

### 2.5. Statistical Analysis

Data were recorded in Microsoft Excel (version 16.78) and analyzed using SPSS (version 27.0; SPSS Inc., Chicago, IL, USA). One-way ANOVA was used to examine the statistical significance of interval scale parameters across groups. For statistical analysis of BCVA in decimal, the one-way ANOVA non-parametric Kruskal–Wallis test was used. Fisher’s exact and chi-square tests were applied to analyze categorical parameters. The threshold for statistical significance was defined as *p*-value < 0.05. Capillary densities are shown as mean ± standard deviation.

## 3. Results

### 3.1. Study Group Characteristics

An amount of 34 hypoxic patients due to various etiologies, including chronic obstructive pulmonary disease (COPD), idiopathic pulmonary fibrosis, pulmonary hypertension, post-COVID-19 lung disease, and restrictive lung disease, and 23 age-matched healthy control subjects were recruited to this study (Supplementary Table S1 and Supplementary Figure S1). Five hypoxic and two control subjects who could not perform the OCT-A test due to severe respiratory illness and physical instability while performing the test, respectively, were excluded from the analysis. In addition, eight hypoxic patients were excluded due to background ocular pathology, primarily diabetic retinopathy. One hypoxic subject with O2 saturation in room air of ≥92% at the exam time was also excluded. The final analysis included 20 patients in the hypoxia group and 21 control subjects. Mean age (61.2 ± 10.2 vs. 59.05 ± 9.4 years old; *p* = 0.487), sex (14% vs. 47.6% males; *p* = 0.146), axial length (23.4932 vs. 24.0238 mm; *p* = 0.179), and intraocular pressure (12.80 vs. 14.43 mmHg; *p* = 0.098) were similar between the two study groups (Table 1). Cardiovascular disease was more prevalent among the hypoxic patients, who also presented a slightly diminished BCVA (0.82 vs. 0.919 decimal; *p* = 0.032; Table 1).

### 3.2. Retinal Analysis

The retina was examined for the vascular and structural characteristics of different slabs and anatomical locations within the macula.

#### 3.2.1. Retinal Thickness

The retinal thickness in multiple macular areas and layers was measured using the ETDRS chart, the ETDRS Retina Thickness v0.3, and the superficial and GCIPL analysis v0.3 algorithms (see Supplementary Figure S2).

The ganglion cell layer (GCL), defined as the layer between the inner margin of the GCL and the inner plexiform layer (IPL), and the superficial retinal slab, defined as the area between the inner limiting membrane (ILM) and the IPL, were significantly thinner for the hypoxic group compared to the control group in the inner nasal, inner superior sections; and the encircling 3 mm area around the fovea (Table 2; see Supplementary Data). These significant differences were observed between hypoxic patients (with and without supplemental O2) and the control group in all sections but not between the two hypoxic subgroups, indicating a real structural change associated with chronic hypoxia rather than acute-on-chronic change.

#### 3.2.2. Retinal Vasculature

The retinal vasculature network was examined for perfusion, capillary density, and FAZ area in multiple anatomical locations within various retinal layers (Table 3) using macular density and superficial GCIPL analysis algorithms. There were no statistical differences between the capillary density of the SCP and DCP in all groups. Importantly, no changes were observed between the two hypoxic subgroups, indicating that acute-on-chronic hypoxia does not affect the inner retinal perfusion in the short term. The FAZ parameters, including raw length, size, and circularity in different foveal slabs, also showed no significant difference between the three subgroups (Table 3).

### 3.3. Optic Nerve Head (ONH) Analysis

The angiography images of the ONH were obtained using 6 × 6 mm scans and analyzed using the ARI network Peripapillary Nerve Fiber Layer Microvasculature Density v0.9 algorithm (see Supplementary Figure S3).

#### 3.3.1. Retinal Nerve Fiber Layer Thickness

Analysis of the structural OCT images of both hypoxic and control groups revealed similar RNFL thicknesses in all ONH regions except for the nasal section area, which was thinner for the control group (mean 75.29 µm) compared to the chronic hypoxic group using supplemental O2 (mean 81.24 µm) and the acute on chronic hypoxic conditions without supplemental O2 (mean 84.37 µm; *p* = 0.036; Table 4).

#### 3.3.2. Peripapillary Microvasculature Characteristics

**Figure 1:**
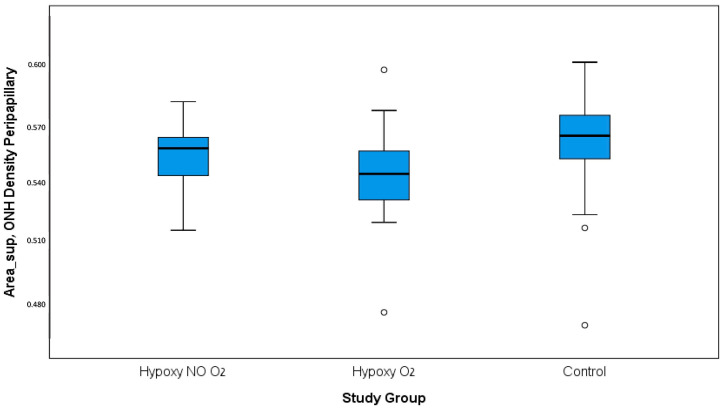
Peripapillary vascular density. A significant difference was found between the groups, with a higher density in the control group. ONH: optic nerve head.

Both peripapillary capillary density and perfusion were examined using the ARI network for all anatomical sections. The analysis using the Kruskal–Wallis test showed that there was a significant difference in the vasculature density in the superior part of the ONH between the control and both hypoxic groups (Figure 1), with a mean density of 0.371 ± 0.03 for the hypoxic group with supplemental O2, 0.383 ± 0.03 for the hypoxic group without supplemental O2 (acute-on-chronic hypoxia), and 0.387 ± 0.03 for the control group (*p* = 0.018). When examining pairwise comparison, a more significant difference was noted, demonstrating a higher capillary density for the control group than the chronic hypoxic group supplemented with O2 (Bonferroni correction, *p* = 0.018).

#### 3.3.3. Choriocapillaries Density

The choriocapillaries were analyzed using one-way analysis of variance (ANOVA) and the Tukey–Kramer comparison test to compare the differences between the three study subgroups. As shown in Table 5 and Table 6, a significant difference was found between the control group and the two hypoxic subgroups in the choriocapillaries count analysis (control—9749.43 ± 2881.6, hypoxic with supplemental O2—12,689.9 ± 1815.1, and hypoxic without supplemental O2—13,073.6 ± 1812.2; *p* < 0.001). A profound difference was also found in the total area of the choriocapillaries between the control (30,979.71 ± 9635.2) and the two hypoxic subgroups with supplemental O2 (44,347.10 ± 10,563.3) and without supplemental O2 (46,984.1 ± 12,822.4) (*p* < 0.01 and *p* < 0.001, respectively). In the percentage area calculation, a meaningful difference was found between the hypoxic group of patients without supplemental O2 (6.2 ± 5.4) and the control group (2.95 ± 0.91) but not with the hypoxic patients with supplemental O2 (4.23 ± 1.0; *p* > 0.05). The mean size of the choriocapillaries did not demonstrate any differences between the study groups.

## 4. Discussion

This prospective study investigated the structural and vascular changes in the retina, optic nerve head, and choroid of patients with chronic and acute-on-chronic hypoxic conditions, compared to age-matched healthy controls, using optical coherence tomography angiography, a powerful imaging technique for detecting pathological alterations in the retina.

Our study found that systemic hypoxia did not appear to induce vascular alterations in the retina’s superficial capillary plexus and deep capillary plexus. This observation was held for both chronic and acute-on-chronic hypoxic conditions, in contrast to a recent study that described reductions in both the SCP and DCP along with an enlarged foveal avascular zone [12]. While the study by Songur et al. examined a homogeneous group of COPD patients, the present study included a more heterogeneous patient population with various underlying etiologies for hypoxia. This difference in patient etiology may have contributed to the discrepant findings. On the one hand, the current study establishes findings regarding hypoxic patients without delving into the specific causes of hypoxia. However, this broad approach may have obscured changes related to the non-hypoxic factors associated with the systemic pathophysiology of COPD.

Additionally, differences between the studies may be attributed to using different OCT-A instruments and analysis algorithms. The previous study did not specify whether the COPD patients required supplemental oxygen throughout the day, as was the case in the current cohort, nor did it mention using oxygen during the OCT-A assessment. If the COPD patients in the earlier study were not well controlled, the hypoxia might have had more significant and severe implications on their vascular bed compared to the patients in the present cohort [12].

Despite previous evidence of altered lung microvasculature in COPD patients, the study found a surprising lack of difference in the retinal vascular densities between the different groups. Yoshimura and colleagues reported that more COPD exacerbations were associated with a lower cross-section area of small pulmonary vessels, suggesting that the vascular changes in the lungs may result from direct damage rather than being solely secondary to hypoxia, as previously proposed [23].

Another possible explanation for the discrepancies between the different studies is the selection bias in this study. Five eligible patients with very severe hypoxic disease were unable to complete the OCT-A exam due to motion artifacts secondary to their lung condition. Additionally, two more patients could not withdraw from their supplemental O2 long enough to undergo the OCT-A test. Excluding these patients with very severe hypoxia may have prevented the characterization of subtle vascular changes. Furthermore, despite severe systemic hypoxia, the relatively preserved vascular network in the retina could be attributed to the augmented vascular autoregulation of ocular blood flow. This is because the retinal oxygen demand is the highest among body organs, as one of the most metabolically active tissues in the body [24,25,26].

Previous research by Fallon et al. demonstrated that normal individuals subjected to hypoxic conditions exhibited increased retinal vascular blood flow [27]. In normal subjects, the larger vessels in the retina were found to autoregulate blood flow in response to changes in oxygen concentration. Specifically, a decrease in blood flow was observed in response to increased oxygen levels, indicating that retinal blood flow increases in hypoxic conditions [27]. This vascular autoregulation in response to hypoxia may also occur in chronic hypoxic patients, potentially explaining why the vascular density in the retinas of our cohort was within normal limits despite their systemic hypoxic condition.

Notably, no significant differences were observed between the two hypoxic cohorts, comprising patients with chronic hypoxia and those with acute-on-chronic hypoxia. This suggests that acute hypoxia does not substantially impact the retinal vascular network.

Different systemic conditions can affect the retinal microvasculature. Sun et al. found that OCT-A parameters changed in response to the Valsalva maneuver, with an increased foveal avascular zone and decreased parafoveal and perfused peripapillary retinal vessel density. The healthy subjects in that study showed these changes due to blood pressure fluctuations. Similarly, the retinal and peripapillary vascular alterations observed may have been normal physiological responses rather than pathological changes expected in chronic hypoxic patients. Moreover, the Valsalva maneuver is known to cause changes in intraocular pressure, which can impact retinal blood flow as detected by OCT-A [28]. Therefore, the lack of significant differences in retinal vascular density between the hypoxic and control groups in the current study may reflect the retina’s robust autoregulatory mechanisms, which can maintain adequate perfusion despite hypoxic conditions. Of note, primary COVID-19 led to transient and reversible retinal microvascular changes in the short term [29], while long-term follow-up demonstrated constant FAZ enlargement as well as a foveal and parafoveal reduction in the capillary density in the DCP [30].

The study by Ozer et al. found reduced blood flow through the extraocular vessels, particularly the ophthalmic artery, in COPD patients compared to normal controls, as measured by duplex ultrasound [31]. However, this blood flow change was not demonstrated in the current study using OCT-A. The discrepancy between the two studies may be attributed to differences in the sensitivity of the imaging techniques, as well as the patient selection, as Ozer et al. examined COPD patients exclusively. In addition, the present study focused on the retinal microvasculature rather than the large vessels examined by ultrasound [31].

The inner retina, composed of the ganglion cell layer and the superficial retinal layers, was thinner in the hypoxic patients compared to the control group in our study. This finding is consistent with a previous report that showed a direct relationship between retinal nerve fiber layer (RNFL) thinning and the severity of chronic obstructive pulmonary disease in all quadrants of the ONH in hypoxic patients [32]. A recent meta-analysis showed a significant reduction in RNFL thickness in patients with severe COPD compared to healthy controls but not for patients with mild/moderate COPD [33]. However, Kocamis and colleagues did not find a difference in RNFL thickness between COPD patients and healthy controls [34]. This finding underscores the complexity of RNFL response under hypoxic conditions, as previous studies have shown mixed results regarding the impact of systemic hypoxia on RNFL thickness. The RNFL is a sensitive structure that may be affected by various systemic and ocular conditions. Previous studies have demonstrated that RNFL thickness can be reduced in conditions like anemia [35], obstructive sleep apnea syndrome (OSAS) [36], inflammation, and migraines [37]. The impact of systemic hypoxia on RNFL thickness remains controversial, as some studies have reported thinning in COPD [38], while others did not find a significant reduction due to hypoxia [34]. The severity, duration, and underlying etiology of the systemic hypoxic state may influence this variable finding. The RNFL thinning observed in one quadrant of the control group compared to both hypoxia subgroups in the current study aligns with this variable condition.

When comparing the ONH vasculature between the different groups, it was apparent that hypoxic patients had a decreased vessel density in the upper part of the ONH compared to healthy controls. One possible explanation for the affected ONH vasculature in contrast to the retinal vascular network may be the exposure to different pressure environments. The vessels behind the lamina cribrosa are exposed to the cerebrospinal fluid pressure, while the vessels anterior to the lamina cribrosa are exposed to the IOP [39,40]. The cerebrospinal fluid pressure may be altered more than the intraocular pressure in chronic hypoxic patients, leading to changes in the ONH vasculature [41].

The choriocapillaries exhibited significant changes in most parameters studied when comparing the hypoxic and control groups, with an even more pronounced difference between the hypoxic patients without supplemental oxygen and the control group. No differences were found when comparing the two hypoxic subgroups. These changes in choriocapillaries had not been previously demonstrated and likely reflect the increased sensitivity of the choroidal vascular network compared to the retinal network due to systemic hypoxia. The lower blood pressure in the choriocapillaries compared to the retinal vasculature may explain their increased vulnerability to blood flow changes. Previous studies using SS-OCT have shown reduced density of choriocapillaries in chronic hypertension patients, and a decrease in choriocapillary damage can lead to secondary damage to the retina and RPE cells [42,43]. It is important to note that while age has been shown to potentially impact the flow deficit in choriocapillaries, the similar ages of the two groups in this study precluded this factor from affecting the results [20,22].

This study has several limitations. The patient cohort was relatively small, as many chronic hypoxic patients have additional comorbidities, particularly diabetes mellitus. Additionally, the hypoxic conditions within the cohort were diverse. Furthermore, the most severely affected patients could not undergo the OCT-A test, potentially excluding those with the highest likelihood of vascular changes.

## 5. Conclusions

This study demonstrates the effect of systemic chronic hypoxia on the RNFL thickness and vasculature, retinal thickness, and choroidal vessel characteristics. In contrast, the retinal vessels appear to have a more resilient protective mechanism that shields them from the effects of systemic hypoxia on the retinal blood supply and vascular structure. The most significant alterations were observed in the choriocapillaries and ONH vasculature, which exhibited reduced vascular density and flow in hypoxic patients compared to controls. Larger-scale studies with larger patient cohorts are needed to elucidate further the vascular changes in the retina and posterior segment associated with systemic hypoxia.

## Figures and Tables

**Table 1:** Baseline demographic characteristics of hypoxic patients and control subjects included in this study. Best-corrected visual acuity; IOP = intraocular pressure; AL = axial length; DM = diabetes mellitus; HTN = hypertension; S/*p* MI = status post myocardial infarction.

	Hypoxia	Control	*p*-Value
Sex	Male = 14 (70%)	Male = 10 (47.6%)	0.146
Age	61.2 ± 10.2	59.05 ± 9.4	0.487
O_2_% saturation	With O_2_^−^ 94% Without O_2_^−^ 88%	95%	
BCVA (decimal)	0.820	0.919	0.032
IOP (mmHg)	12.80	14.43	0.098
AL (mm)	23.4932	24.0238	0.179
Smoking	11 (55%)	3 (14.3%)	0.006
DM	9 (45%)	2 (9.5%)	0.01
Hyperlipidemia	11 (55%)	2 (9.5%)	0.002
HTN	9 (45%)	4 (19%)	0.074
S/*p* MI	5 (25%)	0	0.021
Obesity	6 (30%)	2 (9.5%)	0.13

**Table 2:** Ganglion cell layer and superficial retinal thickness. Retinal thickness in the ganglion cell layer (GCL) to the inner plexiform layer (IPL) and superficial retinal layer from the inner limiting membrane (ILM) to the IPL. The demonstrated sections in the table had statistically significant differences.

Slab	Location	Control	Hypoxic with O_2_	Hypoxic Without O_2_	*p*-Value
GCL (inner GCL—outer IPL)	Inner nasal	94.578	88.069	86.821	0.026
	Inner superior	94.387	89.113	89.294	0.041
	Circular 3 mm	87.582	82.028	82.121	0.036
Superficial slab (ILM-IPL)	Inner nasal	110.601	103.577	102.406	0.042
	Inner superior	114.794	108.368	108.263	0.05
	Circular 3 mm	103.727	97.427	97.410	0.05

**Table 3:** Retinal perfusion and density. Different retinal locations and the corresponding retinal vascular density and perfusion. No statistically significant difference was found between the groups. GCL= ganglion cell layer; FAZ = foveal avascular zone.

Algorithm	Hypoxic with O_2_	Hypoxic Without O_2_	Control	*p*-Value
Average perfusion, GCL slab	0.26519	0.27316	0.26782	0.942
Average perfusion, superficial slab	0.37227	0.37972	0.38088	0.897
Average perfusion, deep retina slab	0.21258	0.20268	0.22053	0.858
Average perfusion, full retina slab	0.39222	0.40146	0.40582	0.794
Average density, GCL slab	12.29328	12.56434	12.70066	0.924
Average density, superficial slab	16.48050	16.70157	17.10652	0.795
Average density, deep retina slab	10.46929	9.98835	10.98792	0.817
FAZ Raw Length, Superficial slab	6.297	2.485	3.905	0.537
FAZ Circularity, Superficial slab	0.653	0.672	0.633	0.800
FAZ Raw Size, Superficial slab	1.390	0.338	0.634	0.497
FAZ Raw Length, full retina slab	5.928	2.462	3.889	0.529
FAZ Circularity, full retina slab	0.641	0.682	0.636	0.699
FAZ Raw Size, full retina slab	1.287	0.338	0.630	0.505

**Table 4:** Peripapillary retinal nerve fiber layer (RNFL) thickness. RNFL thickness comparing hypoxic patients with and without O2 supplementation to the control subjects. In the nasal section, the hypoxic patients had significantly thickern RNFL. GH: Garway Health sectors. RNFL thickness is shown as mean ± standard deviation.

Group		Average	Temporal	Superior	Nasal	Inferior	Average GH	Temporal GH	Superior Tempopral GH	Superior Nasal GH	Nasal GH	Inferior Temporal GH	Inferior Nasal GH
Hypoxic with O_2_ *n* = 20	Mean & SD	83.0 ± 11.3	65.4 ± 15.4	99.8 ± 19.1	66.2 ± 10.9	105.1 ± 13.9	94.5 ± 13.3	73.55 ± 20.7	118.9 ± 23.0	112.9 ± 30.9	81.2 ± 14.3	140.6 ± 24.1	101.5 ± 19.0
Hypoxic without O_2_ *n* = 18	Mean & SD	84.7 ± 10.1	64.5 ± 14.1	103.5 ± 14.3	68.6 ± 9.5	108.0 ± 15.8	97.1 ± 11.7	73.8 ± 19.6	124.0 ± 16.2	117.7 ± 26.07	85.3 ± 12.2	139.2 ± 24.0	103.9 ± 19.0
Control *n* = 21	Mean & SD	81.3 ± 7.8	62.6 ± 8.6	102.2 ± 16.3	62.8 ± 7.2	102.1 ± 15.4	93.2 ± 8.9	71.6 ± 13.6	123.5 ± 28.2	116.7 ± 27.8	75.2 ± 8.6	139.8 ± 22.0	105.9 ± 20.1
*p*		0.554	0.772	0.788	0.162	0.486	0.573	0.916	0.759	0.860	0.036	0.982	0.772

**Table 5:** Choriocapillaries analyses. Four analyses were performed between the three study subgroups, including count, total area, average size, and % area. (SD—standard deviation).

	Count Mean (SD)	Total Area Mean (SD)	Average Size Mean (SD)	% Area Mean (SD)
Control	9749.43 (2881.6)	30,979.71 (9635.2)	100.40 (456.9)	2.95 (0.91)
Hypoxic with O_2_	12,689.9 (1815.1)	44,347.10 (10,563.3)	3.46 (0.3)	4.23 (1.0)
Hypoxic without O_2_	13,073.6 (1812.2)	46,984.1 (12,822.4)	3.5 (0.4)	6.2 (5.4)

**Table 6:** The Tukey–Kramer multiple comparisons test. Four choriocapillaries analyses were performed between the three groups, including count, total area, average size, and % area.

**Count**			
Comparison	Difference	q	*p*-value
Hypoxic without O_2_ vs. Hypoxic with O_2_	383.72	0.6837	*p* > 0.05
Hypoxic without O_2_ vs. Control	3324.2	5.991	*p* < 0.001
Hypoxic with O_2_ vs. Control	2940.5	5.448	*p* < 0.001
**Total area**			
Comparison	Difference	q	*p*-value
Hypoxic without O_2_ vs. Hypoxic with O_2_	2637.1	0.953	*p* > 0.05
Hypoxic without O_2_ vs. Control	16,004	5.854	*p* < 0.001
Hypoxic with O_2_ vs. Control	13,367	5.027	*p* < 0.01
**% Area**			
Comparison	Difference	q	*p*-value
Hypoxic without O_2_ vs. Hypoxic with O_2_	2.014	1.959	*p* > 0.05
Hypoxic without O_2_ vs. Control	3.289	3.235	*p* < 0.01
Hypoxic with O_2_ vs. Control	1.275	1.289	*p* > 0.05
**Average Size**			
Comparison	Difference	q	*p*-value
Hypoxic without O_2_ vs. Hypoxic with O_2_	0.1070	0.001	*p* > 0.05
Hypoxic without O_2_ vs. Control	96.835	1.104	*p* > 0.05
Hypoxic with O_2_ vs. Control	96.942	1.154	*p* > 0.05

## Data Availability

Data will be available upon motivated request to the authors.

## Supplementary Materials

The following supporting information can be downloaded at https://www.mdpi.com/article/10.3390/jcm13226680/s1. Supplementary Table S1. Categorization of Hypoxia Causes within the Study Population; Figure S1: Patient Recruitment and Selection Process for Hypoxic and Control Groups; Figure S2: Retinal diagram for the artificial retinal network analysis. Figure S3: Schematic map of the optic nerve analysis by the artificial retinal network. The reference [44] is cited in the Supplementary Materials.
