# Supplementary material for: Evaluation of Retinal and Posterior Segment Vascular Changes Due to Systemic Hypoxia Using Optical Coherence Tomography Angiography
Source: J Clin Med. 2024 Nov 7;13(22):6680. doi: 10.3390/jcm13226680 (PMC11594346; doi:10.3390/jcm13226680)
Supplement: Supplementary file 1 [file jcm-13-06680-s001.zip › jcm-3256265-supplementary.pdf]

Supplementary Materials

Supplementary Table S1. Categorization of Hypoxia Causes within the Study Population

Condition	Number of Patients
Idiopathic pulmonary fibrosis (IPF)	2
Chronic obstructive pulmonary disease + Idiopathic pulmonary fibrosis (COPD+IPF)	3
Congestive heart failure (CHF)	1
Restrictive lung disease	1
COVID-19	3
Interstitial lung disease (ILD)	2
Chronic obstructive pulmonary disease + Obstructive sleep apnea syndrome (COPD + OSAS)	2
Chronic obstructive pulmonary disease (COPD)	3
Sarcoidosis	1
Obesity hypoventilation syndrome + Heart failure with preserved ejection fraction (OSH + HFpEF)	1
Heart Failure with Preserved Ejection Fraction (HFpEF)	1

Supplementary Figure S1. Patient Recruitment and Selection Process for Hypoxic and Control Groups

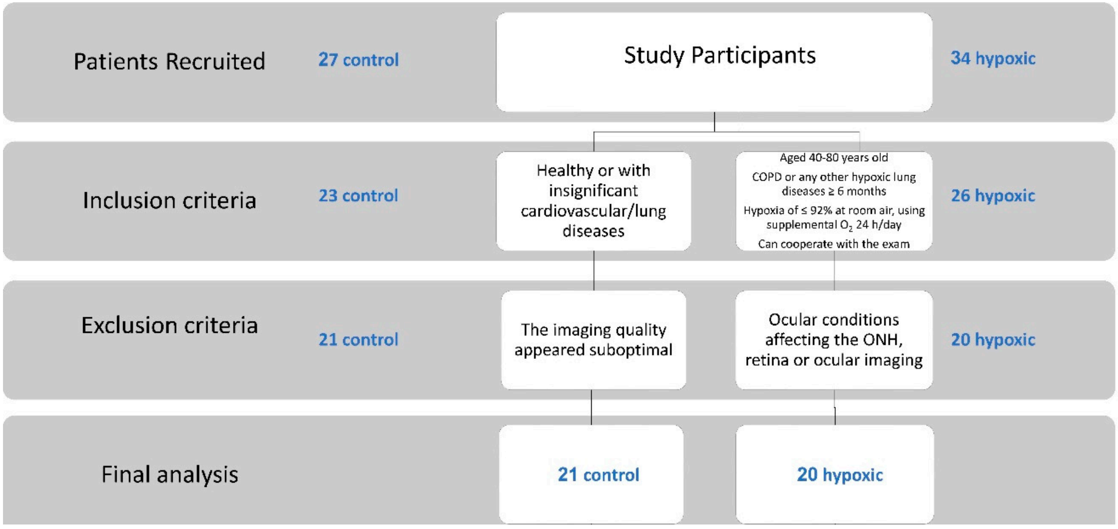

Algorithms in the artificial retinal imaging (ARI network) used for OCT-A scan analysis

Superficial and Ganglion Cell Inner Plexiform Layer (GCIPL) analysis v0.3: This algorithm simultaneously quantifies three aspects of an OCT-A dataset centered on the macula:

Superficial and GCIPL retinal layer thickness

Provides thickness measurements of the superficial layer (defined as the innermost position of the ILM to $10\mu m$ prior to the outermost position of the IPL) and the ganglion cell inner plexiform layer (GCIPL). Layers are generated using multilayer segmentation (MLS), and the results are given in CSV files, including ETDRS, neuro, and radial grids.

Superficial and GCIPL retinal layers vascular density

Measures the perfusion and vascular density. The vascular density is defined as the total length of perfused vasculature per unit area. The vessel density measurement attempts to provide higher sensitivity to the loss of individual capillaries by measuring all vasculature similarly and preventing the larger vessels from obscuring smaller ones.

Foveal avascular zone (FAZ)

This algorithm can identify and quantify the FAZ within the superficial layer. The quantification is of the FAZ's area, perimeter, and circularity index.

Macular Density v0.73

This algorithm quantifies two aspects of an OCT-A dataset: first, the vascular density of the entire retina, including the superficial and deep retinal layers, and second, the foveal avascular zone of the superficial retinal layer.

ETDRS Retinal Thickness v0.3

This algorithm provides total retinal thickness values, measured as the distance between the internal limiting membrane (ILM) and the retinal pigment epithelium (RPE) in different ETDRS sectors. A macula-centered retinal image is divided into nine sectors defined by the ETDRS chart (**Supplementary Figure S2**). The algorithm reports the averaged retinal thickness within each of the sectors.

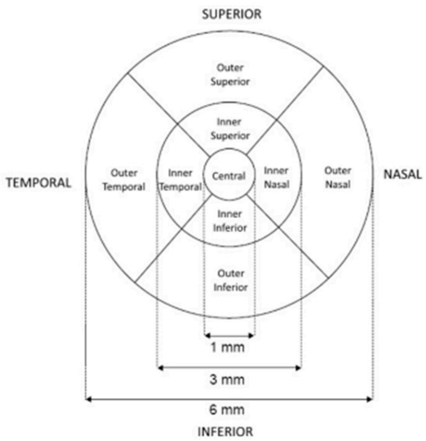

Supplementary Figure S2. Retinal diagram for the artificial retinal network analysis.
Early treatment diabetic retinopathy study (ETDRS) retinal map. The map shows the different retinal areas analyzed for each patient.

Peripapillary Nerve Fiber Layer Microvasculature Density v0.9

This algorithm performs segmentation of the inner limiting membrane (ILM) and retina nerve fiber layer (RNFL), creating radial peripapillary capillary (RPC) vasculature surface, enabling calculation of the capillary density metrics over an annulus centered at the optic disc (**Supplementary Figure S3**). It also quantifies micro-circulation within the peripapillary nerve fiber layer, calculating perfusion, density, and flux index.

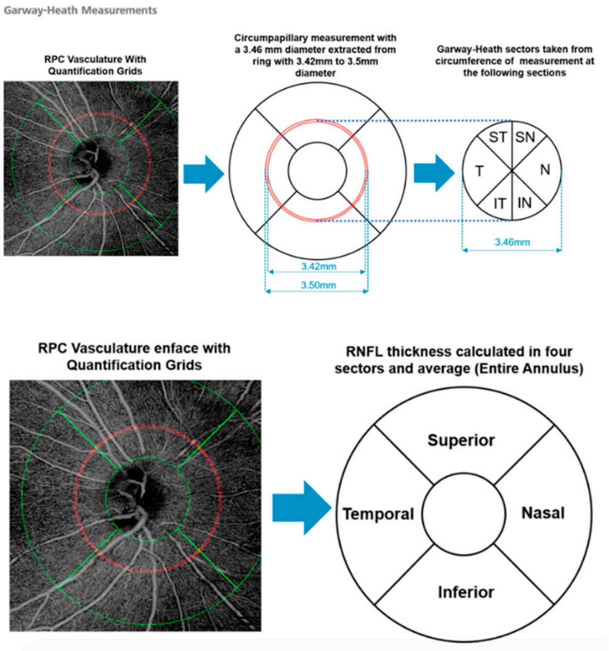

Supplementary Figure S3. Schematic map of the optic nerve analysis by the artificial retinal network. The upper map demonstrates the division of the RNFL in the Garway-Heath sectors. The lower map shows the standard four quadrants RNFL map.

Retinal Vascular Metrics: Capillary Perfusion Density and Capillary Flux Index

Capillary perfusion density represents the total area of perfused microvasculature per unit area in a measured region, with values ranging from 0 to 1. The capillary flux index quantifies the total weighted area of perfused microvasculature per unit area in a measured region.

Choroid Analysis

Choroid analysis was performed according to the protocol by Tiosano et al. [1]. A 10 µm slab, starting 31 µm posterior to the retinal pigment epithelium central line, was used, with manual adjustment as needed. En-face slabs were generated from the angiography and structural data and analyzed using FIJI software (an expanded version of ImageJ software 1.51a, available at Fiji.sc, National Institutes of Health, Bethesda, Maryland, USA).

References

1. Tiosano, L.; Corradetti, G.; Sadda, S.R. Progression of choriocapillaris flow deficits in clinically stable intermediate age-related macular degeneration. *Eye (Lond)* **2021**, *35*, 2991-2998, doi:10.1038/s41433-020-01298-9.
